# Modulation of Tumor Microenvironment to Enhance Radiotherapy Efficacy in Esophageal Squamous Cell Carcinoma by Inhibiting Carbonic Anhydrase IX

**DOI:** 10.3389/fonc.2021.637252

**Published:** 2021-06-25

**Authors:** Pengqin Xu, Yu Zhang, Fanghong Ge, Fuming Zhang, Xia He, Xingya Gao

**Affiliations:** ^1^ The Affiliated Cancer Hospital of Nanjing Medical University, Jiangsu Cancer Hospital, Jiangsu Institute of Cancer Research, Nanjing, China; ^2^ The Affiliated Tumor Hospital of Nantong University, Nantong Tumor Hospital, Nantong, China; ^3^ Department of Physiology, Nanjing Medical University, Nanjing, China

**Keywords:** carbonic anhydrase IX, esophageal squamous cell carcinoma, ethyl N-(4-methylphenyl) sulfonylcarbamate, hypoxia, x-ray irradiation

## Abstract

The radiotherapy outcomes of patients with advanced esophageal squamous cell carcinoma (ESCC) remain poor due to hypoxia. Carbonic anhydrase IX (CAIX) is a membrane-associated enzyme that induces hypoxia, extracellular acidity, and upregulation of hypoxia-related factors in tumor microenvironment, thereby promoting tumor metastasis. CAIX is upregulated in ESCC tissues compared to normal surrounding tissues. In the current study, we aimed to investigate the effect of CAIX inhibition on the modulation of tumor microenvironment and radiotherapy efficacy in ESCC. Higher CAIX expression was correlated with poorer progression-free survival in ESCC patients. Then, the ethyl N-(4-methylphenyl) sulfonylcarbamate (S4) was used to inhibit CAIX expression in ESCC cells and mice xenografts. The pretreatment of ESCC cells with S4 significantly downregulated CAIX expression, decreased intracellular pH, reduced cell viability, resulting in decreased oxygen consumption and more sensitive response to X-ray irradiation. In mice inoculated with ESCC cells, the combination of X-ray irradiation with S4 further improved survival, delayed tumor growth, decreased hypoxia level, exaggerated DNA damage, and increased apoptosis compared with the groups treated solely with S4 or radiotherapy. In conclusion, our study showed that the inhibition of CAIX by S4 treatment altered hypoxic tumor micro-environment, exaggerated DNA damage, increased apoptosis, and thus enhanced radiotherapy efficacy in ESCC. These findings provided a potential therapeutic strategy for patients with resistant ESCC.

## Introduction

Esophageal cancer is the sixth leading cause of cancer-associated death worldwide and esophageal squamous cell carcinoma (ESCC) accounts for approximately 90% of all esophageal cancer cases (1). The annual incidence of ESCC in China is as high as around 280,000 (2). Despite the wide use of radiotherapy for ESCC patients, its therapeutic efficacy is limited due to acquired resistance associated with hypoxia, which reduces the fixation of DNA damage induced by radiation (3). Therefore, continuous efforts are being made to enhance therapy efficacy and thus improve the clinical outcomes of ESCC patients (4).

The tumorigenesis, invasion, and metastasis of ESCC are determined not only by cancer cells but also the tumor microenvironment, a complex, functional niche where tumor progression occurs (5). Hypoxia and extracellular acidity are considered as the key drivers of therapy resistance in solid tumors (6). During radiotherapy, the damage to cancer cells, as well as epithelial cells and blood vessels, leads to the accumulation of radioresistant suppressor cells and the formation of hypoxia area in tumor microenvironment, which subsequently activates immunosuppressive pathways and weakens the antitumor effect of radiotherapy (7). Therefore, a delicate balance between effectively treating the tumor and limiting radiotherapy-induced damage needs to be maintained.

Carbonic anhydrase IX (CAIX) is an enzyme that catalyzes the extracellular conversion of CO_2_ to HCO_3_^−^. It can be activated during hypoxia, leading to aberrant expression of hypoxia-related factors, acidosis of extracellular milieu, and a more alkaline intracellular pH (8). The CAIX inhibitors have been reported to negatively affect tumor growth, invasion, and metastatic dissemination in pre-clinical models, indicating that CAIX may be used as a therapeutic target in cancer treatment (9). It has also been shown that the upregulation of CAIX appears to increase the resistance of cancer cells to radiation (10). Previous studies identified CAIX as a pro-angiogenic factor associated with shorter survival and poor prognosis in ESCC patients (11). High expression of CAIX was also associated with a malignant phenotype in patients with ESCC (12). Birner et al. showed that high expression of CAIX and the hypoxic phenotype in primary esophageal tumors was preserved at least during the formation of lymph node metastases (13). Therefore, we speculated that the expression level of CAIX may be associated with radiosensitivity in ESCC.

In the current study, we investigated the effect of CAIX inhibition on the modulation of tumor hypoxia and radiotherapy efficacy in ESCC both *in vitro* and *in vivo.* We tested the effect of different compounds on the inhibition of CAIX expression, as well as their cytotoxicity. The most potent one was used to examine the effect of CAIX inhibition on ESCC tumor growth and its response to radiotherapy. Our findings revealed that the inhibition of CAIX modulated tumor microenvironment and enhanced efficacy of radiotherapy in ESCC.

## Materials and Methods

### Materials

Anti-CAIX antibody was purchased from Maixin Biotech (Cat No. RAB-0615, Fuzhou, China). The secondary antibody used in Western blot was purchased from Jackson ImmunoResearch (Cat No. 111-035-003, West Grove, USA). BCA assay and MMP assay kits were obtained from Beyotime (Shanghai, China). SDS-PAGE and 10% fetal bovine serum were purchased from Thermo Fisher Scientific (Waltham, USA). The TRIzol extraction kit was obtained from Vazyme Biotech Co., Ltd (Nanjing, China). The CFX Connect™ Real-Time PCR System was purchased from Bio-Rad (Hercules, USA). Acetazolamide (ACTZ), piperidinium acetate (U104), the 3-(4,5-dimethylthiazol-2-yl)-2,5-diphenyltetrazolium bromide (MTT) assay kit, DAPI dye were obtained from Bomei (Heifei, China). The Intracellular pH Detection Kit was purchased from Jining Shiye (Shanghai, China). The Live/Dead Cell Viability Kit was obtained from BestBio (Shanghai, China). The γ-H2AX dye, the antibodies used in DNA damage assessment, TUNEL reagent, anti-hypoxia inducible factor 1 alpha (HIF-1α) antibody, phosphohistone H2AX monoclonal antibody were purchased from Bioss Biotech (Beijing, China). The Apoptosis Detection Kit was obtained from KeyGen Biotech (Nanjing, China).

### Human Tissue Samples

A total of 23 pairs of ESCC and matched adjacent normal tissues were obtained from the Nantong Tumor hospital, Jiangsu, China. The medical records of 56 ESCC patients who had completed radiotherapy between May 2016 and April 2017 were also collected. This study was approved by the Ethics Committee of the Nantong Tumor Hospital and performed in accordance with the Declaration of Helsinki. Written informed consent was obtained from all participants prior to enrollment. ESCC tissue samples were sectioned, stained for anti-CAIX antibody (1:500 dilution) using immunohistochemistry method, and observed under a microscope by two pathologists blinded to the clinical data. Images were captured from five randomly selected fields at 200× magnification. The percentage of positive-stained tumor cells was graded as follows: 1, 0-10% of cells; 2, 11-50% of cells; 3, 51-72% of cells; and 4, >75% of cells stained. The immunostaining intensity was scored as follows: 0, no coloring; 1, slightly yellow staining; 2, yellow or brown-yellow staining; 3, brown staining. The product of the scores of positively-stained cells and immunostaining intensity was calculated. A final score of ≥ 4 was defined as positive CAIX expression, whereas a score of < 4 was defined as negative expression.

### Western Blot

Human tissue samples were homogenized using RIPA buffer containing protease inhibitor and phosphatase inhibitor. The protein concentration was measured by BCA assay. Equal amounts of protein samples were separate on 10% SDS-PAGE, transferred to PVDF membranes, and then incubated with anti-CAIX antibody (1:1000 dilution) at 4°C overnight. After 90-min incubation with a secondary antibody (1:5000 dilution), the blots were visualized and the band density was quantified by Image J software. GADPH was used as an internal control.

### Quantitative Real-Time PCR

Total RNA was extracted from human tissue samples using TRIzol extraction kit and then reverse transcribed to cDNA using a reverse transcription kit. qRT-PCR was performed using a CFX Connect™ Real-Time PCR System. The sequences of the primers were as follows: CAIX forward: 5’-CCAGGGTGTCATCTGGACTG-3’; CAIX reverse 5’-AGGAATTCAGCTGGACTGGC-3’; β-actin forward: 5’-CGTGCGTGACATTAAGGAGAA-3’; β-actin reverse: 5’-GGAAGGAAGGCTGGAAGAGT-3’.

### Cell Culture and Drug Treatment

Human ESCC cell line ECA-109 was purchased from Yuchunbio (Shanghai, China) and cultured in DMEM supplemented with 10% fetal bovine serum, 100 U/mL penicillin, and 100 μg/L streptomycin (10:1:0.1, v/v/v) in a humidified, 5% CO_2_ atmosphere at 37°C until 80% confluency. Hypoxia treatment was performed by maintaining cells in an anaerobic incubator (containing 95% N_2_ and 5% CO_2_) with the oxygen concentration at 1%. All experiments were performed under hypoxic conditions unless otherwise indicated.

ECA-109 cells were plated in 6-well plates at a density of 5×10^5^ cells/well and divided into five groups, and treated with (1) control, (2) DMSO (200 μL), (3) ACTZ (4 mg/mL, 200 μL), (4) ethyl N-(4-methylphenyl) sulfonylcarbamate (S4) (4 mg/mL, 200 μL), and U104 (4 mg/mL, 200 μL). Then cells were treated with or without 200 μL of S4 (1, 2, 3, and 4 mg/mL) or DMSO for 12 h to evaluate the inhibition of CAIX. Groups subjected to hypoxic conditions were added with 2 mL liquid paraffin before culture. The expression of CAIX in each group of cells was measured using a commercially available ELISA kit following the manufacturer’s instructions.

### Assessment of Cellular Cytotoxicity

The MTT assay was performed to assess cellular cytotoxicity induced drugs at different concentrations. ECA-109 cells were plated in 96-well plates at a density of 5×10^3^ cells/well. Twenty-four hours later, cells were incubated with ACTZ, S4, or U104 at a dose of 0, 1, 2, 3, or 4 mg/mL for 24 h. A volume of 200 μL liquid paraffin was added to all groups of cells to ensure hypoxic conditions. Then 20 μL sterile MTT solution was added in each well and incubated at 37°C for 4 h. After adding 200 μL of DMSO, the optical density (OD) was measured at 490 nm using an ELISA analyzer.

### Detection of Intracellular pH

The intracellular pH was measured by the Intracellular pH Detection Kit according to the manufacturer’s protocols. In brief, cells were incubated with BBcell Probe™ solution at 37°C for 30 min. After three washes with phosphate buffer saline (PBS), cells were resuspended in HBSS and then measured using an ELISA analyzer (excitation wavelength: 488-506 nm; emission wavelength: 526 nm).

### Assessment of Cell Migration

Cell migration capacity was assessed by wound healing assay. ECA-109 cells were plated in 6-well plates at a density of 5×10^5^ cells/well. The next day, an artificial straight scratch was made using a sterile pipette tip. After three washes with PBS, cells were treated with or without 200 μL of S4 (4 mg/mL) or DMSO. At 24 and 48 h after treatment, cells were stained using the Live/Dead Cell Viability Kit and observed under a fluorescence microscope at 200× magnification. The relative wound width was calculated as the final scratch width divided by the original scratch width.

### X-Ray Irradiation

ECA-109 cells were plated in 96-well plates and treated with or without 200 μL of S4 (4 mg/mL) for 12 h under hypoxic conditions. Then cells were irradiated at various doses (2, 4, and 8 Gy) by an X-ray linear accelerator (dose rate = 300 cGy/min) in a 20 cm × 20 cm radiation field at a distance of 100 cm at room temperature. After further incubation for 12 h, the expression of CAIX in all groups of cells was measured by ELISA. The X-ray linear accelerator was provided by Siemens (Cat No. ONCOR, Munich, Germany).

### Assessment of Mitochondrial Membrane Potential

The MMP of ECA-109 cells was measured using the JC-1 dye from an MMP assay kit according to the manufacturer’s instructions. Briefly, ECA-109 cells were treated with or without 200 μL of DMSO or S4 (4 mg/mL) under hypoxic conditions followed by X-ray irradiation at 0 or 4 Gy. The Control + RT (-) group remained untreated. Then cells were incubated with 500 μL culture medium plus 500 μL JC-1 at 37°C for 20 min and then observed under a fluorescence microscope. In cells with high MMP, JC-1 spontaneously formed aggregates with intense red fluorescence, while in cells with low MMP, JC-1 monomers exhibited green fluorescence.

### Assessment of DNA Damage

The extent of DNA damage was expressed as the percentage of nuclei positively stained for γ-H2AX, an indicator of DNA double-strand breaks. ECA-109 cells were treated with 200 μL of PBS or S4 (4 mg/mL) under hypoxic conditions followed by X-ray irradiation at 0 or 4 Gy. The control group remained untreated. 12 h later, cells were fixed with 4% PFA solution for 20 min, washed with PBS, and then added with phosphohistone H2AX monoclonal antibody (1:500 dilution) at 4°C overnight. The next day, cells were incubated with a secondary antibody (1:500 dilution) for 2 h, stained for DAPI, and observed under a fluorescence microscope.

### Apoptosis Assay

ECA-109 cells were treated with 2 mg/mL S4 (or DMSO) and irradiated with X-ray at 0 or 4 Gy. Apoptotic cell death was analyzed using an Apoptosis Detection Kit at 2-h post-irradiation. Cells were trypsinized, centrifuged, and resuspended with 400 μL 1× Annexin V buffer. After 5-min incubation with 5 μL Annexin V-FITC and 5 μL propidium iodide at 2-8°C in the dark, flow cytometry analysis was performed.

### Colony Formation Assay

Cells were seeded into 6-well flat-bottom plates at a density of 4000 cells/well. Then, cells were treated with 200 μL of S4 (2 mg/mL) or PBS for 4 h. After removing the supernatant, cells were irradiated with 4 Gy X-ray and incubated for additional 12 days to form cell clones. Subsequently, cells were fixed with methanol, stained with 0.1% crystal violet, and then counted under a dissecting microscope. Clones were defined as groups of more than 50 cells.

### Animal Study

An ESCC xenograft model was established by subcutaneously inoculating 100 μL of ECA-109 cells (3×10^7^ cells/mL) into the right forelimb of male BALB/c nude mice (age: 6-8 weeks old; weight: 18-20 g). A tumor volume of 100 mm^3^ indicated that the model was successfully established. All animals were housed in an environment-controlled room with a temperature of 22 ± 2°C, a humidity of 45%, and 12-h light-dark cycle. They had *ad libitum* access to water and food. This study was approved by the Ethics Committee of the Nantong University, and performed in accordance with the Guide for the Care and Use of Laboratory Animals.

Twenty mice inoculated with ECA-109 cells were randomly divided into four groups (n=5 per group): control, S4, PBS+RT, and S4+RT. Mice in the PBS+RT and S4+RT groups were irradiated with X-ray at 4 Gy every four days for four times. Mice in the S4+RT group were also injected with S4 (10 mg/day) *via* the tail vein 2 h before each irradiation. The S4 group was administered with S4 (10 mg/day) every four days for four times without irradiation. The control group was injected with 0.1 mL PBS following the same procedure. The survival rate was recorded at the end of each week. The tumor volume was examined every four days for 28 days using the formula: V=0.5×a×b^2^ (a, the greatest diameter; b, the shortest diameter). Tumor samples were collected and weighed at the end of the study (death or at 24 h after the last irradiation). Heart, liver, spleen, lung, and kidney tissues were also harvested at days 1, 14, and 28 following treatment. All tissue samples were examined for histopathological changes by hematoxylin and eosin (H&E) staining.

Sectioned tumor tissues from all groups of mice were stained for CAIX (1:500 dilution) using immunohistochemistry method. Tissue samples were also stained with TUNEL reagent and anti-hypoxia inducible factor 1 alpha (HIF-1α) antibody for the detection of apoptosis and hypoxia, respectively. The percentage of TUNEL-positive area and the relative expression of HIF-1α were measured under a fluorescence microscope. The DNA damage in tumor tissues was determined by the number of γ-H2AX foci. Fixed tumor tissue sections were incubated with phosphohistone H2AX monoclonal antibody (1:500 dilution) at 4°C overnight followed by 2-h incubation with a goat anti-rabbit secondary antibody (1:500 dilution). The nuclei were stained for DAPI. The slides were observed under a fluorescence microscope.

### Statistical Analysis

Data are shown as mean ± standard deviation from at least three independent experiments, each performed in triplicate. Data were analyzed by software SPSS (version 16.0). In cell culture experiment and animal study, *t*-test and two-way analysis of variance (ANOVA) were used to evaluate statistical significance. When analyzing clinical samples, Mann-Whitney test and Chi-squared test were used to compare continuous and categorical variables, respectively. The Kaplan-Meier method was used to analyze progression-free survival (PFS). A value of *P*<0.05 was considered statistically significant. * *P*<0.05, ** *P*<0.01.

## Results

### The Expression of CAIX Is Upregulated in ESCC Tissues

**Figure 1:**
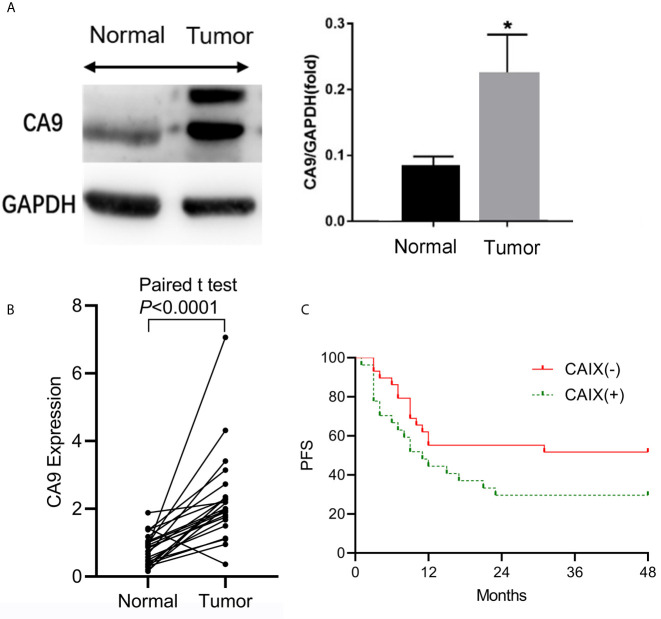
The expression of CAIX in ESCC tissues and its clinical significance. Twenty-three pairs of ESCC and matched adjacent normal tissue samples were obtained for analysis. **(A)** The protein and mRNA expression levels of CAIX were measured by Western blot and qRT-PCR, respectively. **(B)** ESCC tissue samples were stained for CAIX using immunohistochemistry method. **(C)** The Kaplan-Meier survival analysis of ESCC patients with positive (n=27) or negative (n=29) expression of CAIX. *P < 0.05.

To understand the role of CAIX in the ESCC tumor microenvironment, we compared the expression of CAIX between ESCC and paired adjacent normal tissues obtained from 23 ESCC patients. Compared to adjacent noncancerous tissue samples, CAIX was upregulated in ESCC tissues at both protein (**Figure 1A**) and mRNA (**Figure 1B**) levels. To further explore the role of CAIX in ESCC treated with radiotherapy, the medical records of 56 ESCC patients who had completed radiotherapy were collected. Their demographic and clinicopathological characteristics are shown in **Table 1**. The immunohistochemistry staining of ESCC tissue samples revealed that 48.2% (27/56) of the tumors were positively stained for CAIX. However, the positive expression of CAIX in ESCC tissues was not significantly associated with the gender, age, tumor size, lesion location, and lymphatic metastasis in ESCC patients. The Kaplan-Meier survival analysis of 56 ESCC patients showed that positive CAIX expression was correlated with significantly poorer PFS compared with negative expression (**Figure 1C**). These findings suggest that CAIX may play an oncogenic role in the progression of ESCC.

**Table 1:** Association between CAIX expression and clinicopathological characteristics of ESCC patients.

Parameter	Number of cases	CAIX		χ2	P
		Negative	Positive		
**Number of case**	56	29	27		
**gender**					
**male**	31	17	14	0.2592	0.6106
**Female**	25	12	13
**Age (y)**					
**≤69**	27	13	14	0.2763	0.5991
**>69**	29	16	13
**Tumor size (cm)**					
**≤4.7**	29	15	14	0.0000	0.9924
**>4.7**	27	14	13
**Lesion location**					
**Upper esophagus**	16	7	9	0.7561	0.6852
**Mild esophagus**	37	20	17
**Lower esophagus**	3	2	1
**Lymphatic metastasis**					
**No**	32	19	13	1.7224	0.1894
**Yes**	24	10	14

### The Process of CAIX Inhibitor

**Figure 2:**
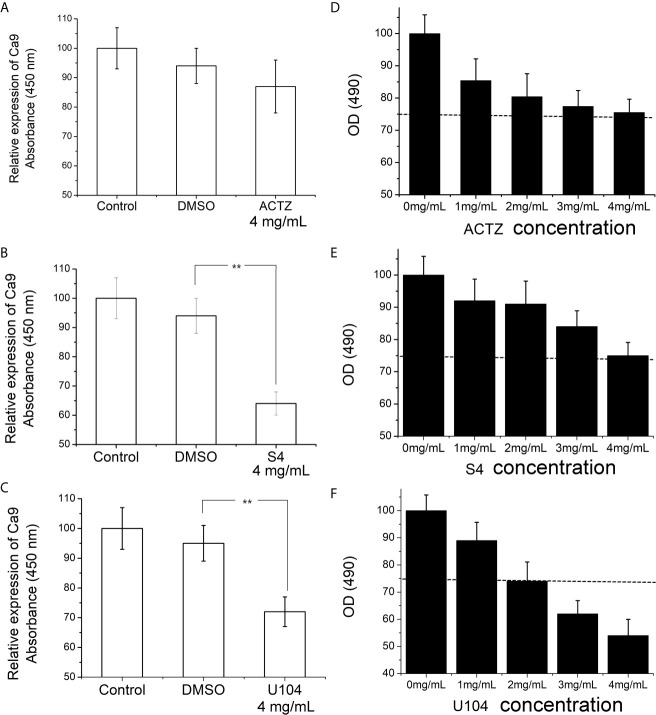
Effect of ACTZ, S4, and U104 treatment on CAIX expression and cell viability in ECA-109 cells. ECA-109 cells were treated with or without ACTZ (4 mg/mL), S4 (4 mg/mL), U104 (4 mg/mL), or DMSO for 12 h under hypoxic conditions. **(A–C)** The expression of CAIX in each group of cells was measured by ELISA. **(D–F)** Cell viability results of ECA-109 cells treated with ACTZ, S4, U104 at various doses (0, 1, 2, 3, and 4 mg/mL) for 24 h. **P < 0.01.

To choose the most potent inhibitor of CAIX expression in ESCC cell line, we treated ECA-109 cells with three sulfonamides (ACTZ, S4, and U104) at 4 mg/mL for 12 h under hypoxic conditions. Cells incubated with S4 and U104 showed significantly downregulated CAIX compared to the control and DMSO-treated groups (*P*<0.01), whereas ACTZ treatment did not alter the expression of CAIX in ECA-109 cells (**Figures 2A–C**). We further assessed the cytotoxicity of ACTZ, S4, and U104 on ECA-109 cells at different concentrations. S4 exhibited better cell compatibility compared to U104 and therefore was used to inhibit CAIX expression in this study (**Figures 2D–F**). S4 at a concentration of 4 mg/mL potently inhibited CAIX expression in ESCC cells without cytotoxicity, therefore, was used in subsequent experiments.

### S4 Regulated Tumor Cell Movement *In Vitro*

**Figure 3:**
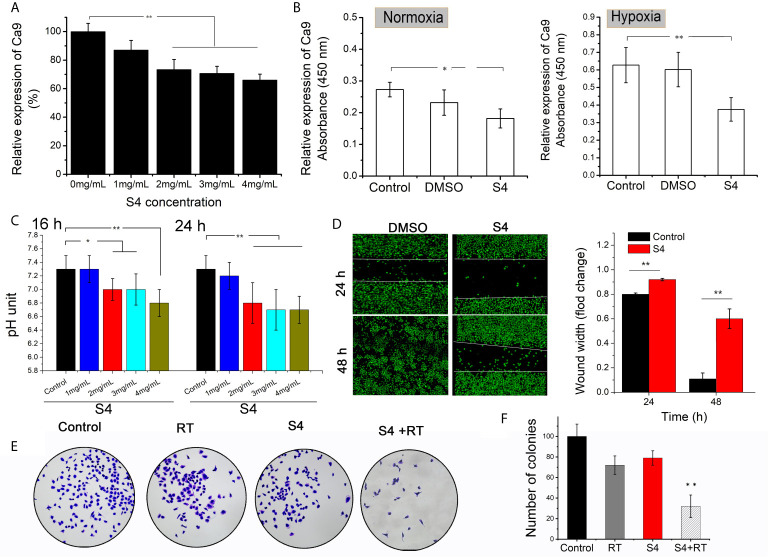
Effect of S4 treatment on CAIX expression, intracellular pH, and migration capacity of ECA-109 cells. **(A)** ECA-109 cells were treated with S4 at various doses (0, 1, 2, 3, and 4 mg/mL) for 12 h under normoxic conditions. The expression level of CAIX in each group of cells was measured by ELISA. **(B)** ECA-109 cells were incubated with or without S4 (4 mg/mL) or DMSO for 12 h under normoxic or hypoxic conditions. The expression level of CAIX was assessed by ELISA. **(C)** ECA-109 cells were treated with or without S4 at different concentrations (1, 2, 3, and 4 mg/mL) under hypoxic conditions. The intracellular pH was measured by at 16-h or 24-h post-treatment. **(D)** Transwell assay was performed to evaluate the migration capacity of ECA-109 cells treated with or without S4 (4 mg/mL) for 24 or 48 h. **(E, F)** Cells were treated with or without 200 μL of PBS or S4 (2 mg/mL) for 4 h and then irradiated with 4 Gy X-ray and incubated for 12 days to form cell clones. Subsequently, cells were stained with 0.1% crystal violet and the number of colonies were counted under a microscope. *P < 0.05, **P < 0.01.

The protein expression of CAIX in ECA-109 cells was significantly downregulated by S4 treatment starting at a dose of 2-4 mg/mL (**Figure 3A**). The inhibitory effect of S4 on CAIX expression was more robust when cells were cultured under hypoxic conditions (**Figure 3B**). Moreover, treatment of ECA-109 cells with 2-4 mg/mL S4 resulted in significantly decreased intracellular pH values compared to the control group at both 16 and 24 h post-treatment (**Figure 3C**). Based on these results, cells subjected to S4 treatment were cultured in the presence of 4 mg/mL S4 under hypoxic conditions in the following experiments. The effect of S4 treatment on the migration capacity of ECA-109 cells was evaluated by Transwell assay. Compared to the control group, S4 treatment significantly impeded the migration of ECA-109 cells at both 24 and 48 h (**Figure 3D**). Moreover, cells subjected to both S4 treatment and 4 Gy X-ray irradiation showed significantly decreased number of colonies, while those treated with X-ray irradiation or S4 alone showed no significant difference in colony forming capacity in comparison to control cells (**Figures 3E, F**). These results showed that S4 treatment suppressed CAIX expression, decreased intracellular pH, and inhibited cell migration in ECA-109 cells.

### S4 Treatment Enhances the Efficacy of X-Ray Irradiation on ECA-109 Cells

**Figure 4:**
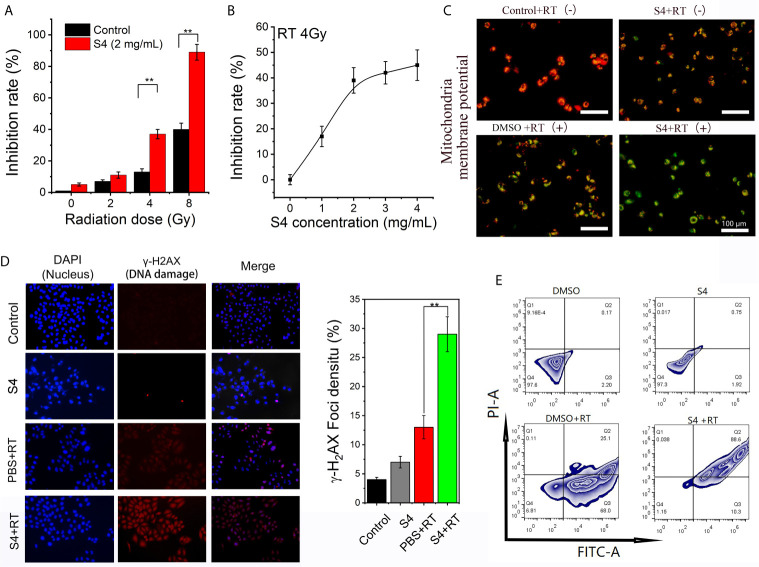
Effect of S4 treatment on the efficacy of X-ray irradiation on ECA-109 cells. **(A)** ECA-109 cells were treated with or without S4 (4 mg/mL) for 12 h under hypoxic conditions followed by X-ray irradiation at 0, 2, 4, or 8 Gy. After 12-h incubation, the expression of CAIX in all groups of cells was measured by ELISA. **(B)** ECA-109 cells were treated with varied doses of S4 (0, 1, 2, 3, and 4 mg/mL) for 12 h under hypoxic conditions followed by X-ray irradiation at 4 Gy. After 12-h incubation, the expression of CAIX in all groups of cells was measured by ELISA and the inhibition rate curve was plotted. **(C)** ECA-109 cells were treated with S4 (4 mg/mL) or DMSO for 12 h under hypoxic conditions followed by X-ray irradiation at 0 or 4 Gy. The Control + RT (-) group remained untreated. The S4 + RT (-) group was treated with S4 (2 mg/mL) only. The MMP was measured using the JC-1 dye. Red fluorescence indicates high MMP, whereas green fluorescence indicates low MMP. **(D)** ECA-109 cells were treated with S4 (4 mg/mL) or PBS for 12 h under hypoxic conditions followed by X-ray irradiation at 0 or 4 Gy. The control group remained untreated. Cells were stained for DAPI (marker of nuclei, blue) and γ-H2AX (marker of DNA damage, red). The extent of DNA damage was expressed as the density of γ-H2AX foci in cell nuclei. **(E)** ECA-109 cells were treated with S4 (4 mg/mL) or DMSO for 12 h under hypoxic conditions followed by X-ray irradiation at 0 or 4 Gy. The apoptotic cell death was analyzed by flow cytometry. **P < 0.01.

To investigate whether S4 treatment would affect the efficacy of radiotherapy on ESCC, we first examined the inhibitory effect of X-ray irradiation on the expression of CAIX in S4-pretreated ECA-109 cells. S4 treatment significantly enhanced the inhibitory effect of irradiation (4 and 8 Gy) on CAIX expression (**Figure 4A**). Next, we examined the inhibitory effect of X-ray irradiation (4 Gy) in cells pre-treated with different doses of S4, and found that the inhibitory effect of irradiation on CAIX expression was increased with S4 pretreatment in a dose-dependent manner (**Figure 4B**). Therefore, X-ray irradiation at a dose of 4 Gy was used for the following experiments. The combination of S4 with X-ray irradiation further decreased the MMP of ECA-109 cells compared to the groups treated with S4 or X-ray irradiation alone (**Figure 4C**). RT treated cells pretreated with S4 also showed a significantly higher density of γ-H2AX foci in the nuclei (about 1.9-fold), indicating exaggerated DNA damage compared to the group without S4 treatment (**Figure 4D**). Furthermore, cells administered with S4 followed by X-ray irradiation also showed increased apoptotic cell death compared to the groups received S4 treatment or X-ray irradiation alone (**Figure 4E**). The above findings implied that S4 treatment enhanced the efficacy of X-ray irradiation on ECA-109 cells.

### S4 Treatment Enhances the Efficacy of Radiotherapy on ESCC *In Vivo*

To examine the effect of S4 treatment *in vivo*, we established an ESCC xenograft model using BALB/c mice *via* subcutaneous inoculation with ECA-109 cells. Mice were randomly divided into four groups and receive different treatments for 28 days: control (treated with PBS vehicle only), S4 (treated with S4 treatment alone), PBS+RT (treated with PBS vehicle plus X-ray irradiation at 4 Gy), and S4+RT (treated with S4 treatment plus X-ray irradiation at 4 Gy).

**Figure 5:**
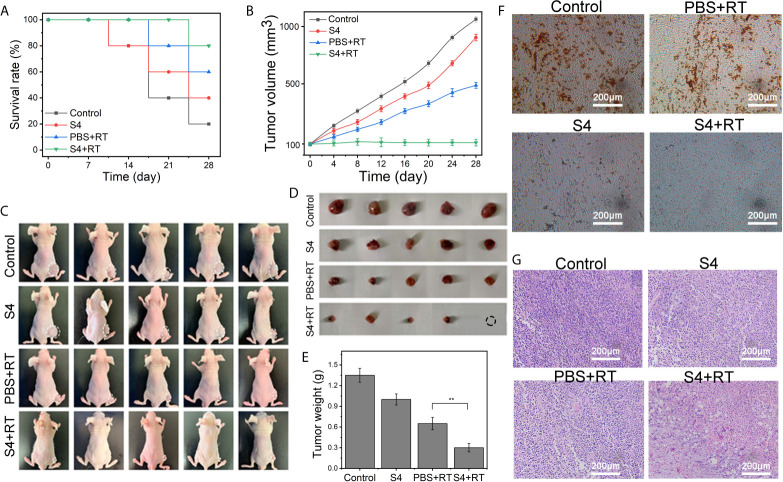
Effect of S4 treatment plus irradiation on tumor growth in ESCC xenograft model. An ESCC xenograft model was established in male BALB/c nude mice *via* subcutaneous injection with ECA-109 cells. Mice were randomly divided into four groups (n=5 per group): control (PBS vehicle), S4 (S4 treatment), PBS+RT (PBS vehicle plus X-ray irradiation at 4 Gy), and S4+RT (S4 treatment plus X-ray irradiation at 4 Gy). **(A)** The survival rate was recorded at the end of each week for four weeks. **(B)** Tumor volume was examined every four days for 28 days. **(C)** Images of each animal at the end of the study (death or at day 28 post-treatment) were shown. **(D, E)** Tumor samples were collected and weighed immediately after death or at day 28 post-treatment. Tumor tissue samples were stained for **(F)** CAIX using immunohistochemistry and **(G)** H&E (pathological changes). **P < 0.01.

The survival rates of the control, S4, PBS+RT, and S4+RT groups at day 28 were 20%, 40%, 60%, and 80%, respectively (**Figure 5A**). Because of hypoxia, The combination of PBS+RT (treated with PBS vehicle plus X-ray irradiation at 4 Gy) slightly decreased tumor volume compared with the groups administered with the control (treated with PBS vehicle only). Notably, the combination of S4 with radiotherapy also markedly decreased tumor volume compared with the groups administered with S4 or X-ray irradiation alone (**Figure 5B**). The images of each animal (**Figure 5C**) and tumor sample (**Figure 5D**) are shown. Mice administered with both S4 and X-ray irradiation also showed significantly decreased tumor weight compared to the group treated with radiotherapy alone (**Figure 5E**).

**Figure 6:**
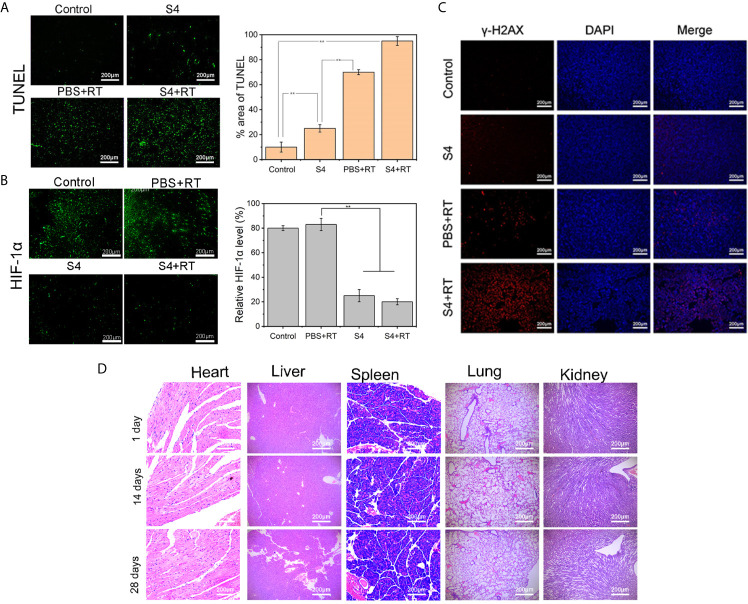
Effect of S4 treatment plus irradiation on apoptosis, hypoxia, and DNA *in vivo.* Mice inoculated with ECA-109 cells were divided into four groups (n=5 per group): control (PBS vehicle), S4 (S4 treatment), PBS+RT (PBS vehicle plus X-ray irradiation at 4 Gy), and S4+RT (S4 treatment plus X-ray irradiation at 4 Gy). Tumor samples were collected at the end of the study (death or at day 28 post-treatment). Tumor samples were sectioned and stained for **(A)** TUNEL reagent (apoptosis), **(B)** HIF-1α (hypoxia), and **(C)** γ-H2AX and DAPI (DNA damage). **(D)** Heart, liver, spleen, lung, and kidney tissues were harvested from the S4+RT group at days 1, 14, and 28 following treatment and examined for histopathological changes by H&E staining. **P < 0.01.

The immunohistochemistry staining of CAIX expression in tumor tissues showed that both the S4 and S4+RT groups had much less brown-stained cells compared to the Control and PBS+RT groups, and the weakest staining was observed in mice treated with both S4 and X-ray irradiation (**Figure 5F**). The H&E (**Figure 5G**) and TUNEL (**Figure 6A**) staining showed that mice treated with both S4 and radiotherapy had significantly more apoptotic cells compared to other groups. The analysis of HIF-1α expression in tumor tissues suggested that S4 significantly decreased about 2.5-fold in the level of hypoxia in ESCC tumors treated with radiotherapy (**Figure 6B**).

Further examination on the expression of γ-H2AX in tumor cell nuclei demonstrated that the combination of S4 with radiotherapy exaggerated DNA damage in ESCC xenograft compared to the groups treated with S4 or X-ray irradiation alone (**Figure 6C**).

Additionally, no long-term toxicity in the heart, liver, spleen, lung, and kidney was observed in mice administered with S4 and X-ray irradiation (**Figure 6D**). Taken together, these results indicated that S4 treatment enhanced the efficacy of radiotherapy on ESCC progression *in vivo* by inhibiting the expression of CAIX and modulating tumor microenvironment.

## Discussion

Despite the increasing effectiveness and precision of radiotherapy, the survival outcomes and local control of patients with advanced ESCC remain poor (14). In this study, we identified CAIX as a key regulator of radiosensitivity in ESCC. The inhibition of CAIX by S4 treatment altered tumor microenvironment and thus enhanced radiotherapy efficacy in both ESCC cells and xenografts.

CAIX is a hypoxia-induced, cell-surface glycoprotein that mostly confined to the epithelia of digestive organs and rarely expressed in non-cancerous tissues (15). It acts as a key regulator of intracellular pH during tumorigenesis through its catalytic activity (16). CAIX is also implicated in cancer progression *via* mediating the adhesion of cancer cells to vessels (17). Previous studies found that CAIX directly participates in a multitude of intratumoral acidosis-related oncogenic events, such as the upregulation of the key drivers of tumorigenesis (e.g. lactate dehydrogenases, glucose transporters, and monocarboxylate transporters) and the induction of extracellular matrix components (e.g. collagens and matrix metallopeptidases) (18–20). Moreover, a meta-analysis reported that CAIX expression was significantly associated with advanced progression and poor prognosis in multiple human cancers, including head and neck, breast, and colorectal cancer (21). Consistently, we found that CAIX was upregulated in ESCC tissues compared to adjacent normal tissues and higher CAIX expression was correlated with poorer PFS in ESCC patients. Previous data revealed that CAIX can be activated during hypoxia, contributing to the maintenance of an alkaline intercellular pH in tumor cells and the upregulation of proteins related to epithelial-mesenchymal transition, thereby facilitating tumor growth, invasion, and migration (22). Moreover, CAIX has been identified as a key mediator of hypoxia-induced stress response in cancer cells (23). A study by Drenckhan et al. showed that selective inhibition of CAIX and a CAIX knockdown effectively inhibited proliferation and migration of esophageal carcinoma tissues (24). In this study, S4 was selected to inhibit CAIX expression in ESCC cells under hypoxic conditions. Compared to the vehicle-treated group, cells incubated with S4 showed significantly downregulated CAIX expression, decreased intracellular pH, and reduced migration capacity.

During radiation response, accumulated free radicals and intermediate ions induce DNA damage in the forms of mitochondrial DNA lesions and strand breaks (25). However, some tumors may acquire resistance to radiotherapy, which has become a major clinical challenge in cancer treatment. Both hypoxia and acidosis contribute to increased radioresistance in tumors (26). Under hypoxic conditions, DNA radicals are reduced to its original form, which inhibits the generation of strand breaks (27). It has also been proposed that the mechanisms of hypoxic radioresistance involving the accumulation of HIF-1α and its dimerization with HIF-1β, which enhances the transcription of hundreds of carcinogenic genes and thus confers radioresistance (28). Acidosis has also been reported to enhance radioresistance by modulating the intracellular levels of HIF-1α (29). The alterations in tumor microenvironment lead to reduced fixation of radiation-induced DNA damage, delayed G2/M-phase arrest, and increased cell survival (30). Radioresistance also causes aberrant expression of mitochondrial protein, increases MMP, and inhibits apoptosis in cancer cells (31). As a sensitive responder to hypoxia and a key regulator of acidosis, CAIX has been shown to protect tumor cells against irradiation damage by maintaining an alkaline intracellular pH and decreasing radiation-induced apoptosis (32). In the current study, we showed that pretreatment of ESCC cells with S4 significantly enhanced the inhibitory effect of irradiation on CAIX expression, decreased the MMP, exaggerated DNA damage, and increased apoptotic cell death compared to cells received S4 treatment or X-ray irradiation alone.

Whether the combination of radiotherapy with S4 would achieve better therapeutic effects against the development of ESCC compared to either of them administered alone was further evaluated in xenografts. Our results demonstrated that mice treated with both S4 and X-ray irradiation had better survival, delayed tumor growth, and increased apoptosis compared to the groups administered with S4 or radiotherapy alone. HIF-1α is the oxygen-dependent subunit of hypoxia inducible factor 1, which plays an important role in regulating intracellular pH, invasion, and migration of cancer cells under hypoxic conditions (33). As the major mediator of the adaptability of tumor cells to hypoxia, HIF-1α is constitutively activated in a broad spectrum of solid tumors, such as gastric cancer, cervical cancer, and breast cancer (34–36). A high HIF-1α expression has also been considered as a contributor to radioresistance *via* increasing the ability of DNA repair, inhibiting apoptosis, and mediating the reprograming of energy metabolism in tumor cells (37). In ESCC patients, a upregulated HIF-1α expression is associated with lymph node metastasis and resistance to radiotherapy (38). Here, we showed that S4 treatment significantly decreased the expression of HIF-1α in ESCC tumors treated with radiotherapy. In addition, the combination of S4 with radiotherapy also exaggerated DNA damage in ESCC xenografts compared to the animals treated solely with S4 or radiotherapy.

## Conclusion

In conclusion, this study reported that the CAIX was positivity correlated with poor prognosis and radioresistance in ESCC. The inhibition of CAIX by S4 treatment altered tumor microenvironment, exaggerated DNA damage, increased apoptosis, and thus enhanced radiotherapy efficacy in ESCC. Our findings provided a potential therapeutic strategy for patients with resistant ESCC.

## Data Availability Statement

The original contributions presented in the study are included in the article/supplementary material. Further inquiries can be directed to the corresponding authors.

## Author Contributions

XH, XG, and PX conceived and designed the experiments. PX, YZ, FG, and FZ performed the experiments. YZ analyzed the data. PX wrote the paper. All authors contributed to the article and approved the submitted version.

## Funding

This work was supported by Social Development Key Project of Jiangsu Science and Technology Department (Grant number BE2019756), and Nantong Municipal Commission of Health and Family Planning (Grant number WKZL2018046).

## Conflict of Interest

The authors declare that the research was conducted in the absence of any commercial or financial relationships that could be construed as a potential conflict of interest.
